# Comparison of *Chlamydia trachomatis* infection among infertile and fertile women in Ahvaz, Iran: A case-control study

**Published:** 2017-11

**Authors:** Fatemeh Joolayi, Tahereh Navidifar, Razieh Mohammad Jaafari, Mansour Amin

**Affiliations:** 1 *Department of Microbiology, School of Medicine, Ahvaz Jundishapur University of Medical Sciences, Ahvaz, Iran.*; 2 *Reproductive Health Promotion Research Center, Ahvaz Jundishapur University of Medical Sciences, Ahvaz, Iran.*; 3 *Infectious and Tropical Diseases Research Center, Health Research Institute Ahvaz Jundishapur University of Medical Sciences, Ahvaz, Iran.*

**Keywords:** C. Trachomatis, Infertility, PCR, IgM, IgG

## Abstract

**Background:**

*Chlamydia trachomatis* (*C. trachomatis*) is the main cause of bacterial sexually transmitted infections. In women, this infection can lead to tubal infertility.

**Objective:**

In this study we investigated *C. trachomatis* among infertile and fertile women with both polymerase chain reaction (PCR) and ELISA methods in Ahvaz, Iran.

**Materials and Methods:**

This case-control study was conducted at the Infertility Clinic of University Jahad, Ahvaz, Iran from January to August 2017. A total of 225 vaginal swabs and blood samples (100 infertile and 125 fertile women) were collected. Detection of *C. trachomatis* DNA was performed from vaginal swabs by amplification of MOMP gene. Also, anti *C. trachomatis* immunoglobulin M (IgM) and immunoglobulin G antibodies in the serum samples were recognized by enzyme-linked immunosorbent assay (ELISA).

**Results:**

Results showed that, 6 (6%) infertile and 2 (1.6%) fertile women were positive for IgM (p=0.21). Also, PCR was positive for *C. trachomatis* infection in 5 infertile (5%) and 2 fertile women (1.6%) (p=0.35). We did not find any seropositive immunoglobulin G in both groups.

**Conclusion:**

In this study, no significant difference was found between fertile and infertile groups for *C. trachomatis* infection. Also, the correlation between IgM and PCR results revealed a relatively strong agreement and seems both PCR and IgM assays are appropriate for the accurate diagnosis of* C. trachomatis* infections.

## Introduction

Chlamydia trachomatis is one of the main causes of bacterial sexually transmitted infections (1). According to World Health Organization, each year approximately 101 million chlamydial infections are documented worldwide (2). *Chlamydia* infections in symptomatic patients have an incubation period between 1-3 wk with non-specific symptoms such as abnormal vaginal discharge, intermenstrual bleeding, dysuria or pyuria (3). This infection is asymptomatic in about 70% of infected women and 50% of infected men and for this reason; it remains undiagnosed and can develop complications (4). The lower genital tract infections due to C. trachomatis are more common in women less 25 yr old compare to women over 25 yr old (13.5% vs. 3.3%) (5). Other risk factors associated with this infection are unmarried status, nulliparity, black race and poor socioeconomic condition (6). The women, who carry C. trachomatis asymptomatically, are considered as the potential sources of transmission to their partners (7). In women, this hidden infection can led to pelvic inflammatory disease (PID), which then cause tubal infertility. Chlamydial PID is preventable if antibiotic treatment is recommended on time (8, 9). 

Since the treatment of PID and infertility due to C. trachomatis has high financial costs, expansion of the screening programs for detecting asymptomatic women is essential. The main aims of these programs are early detection and treatment of uncomplicated lower genital tract infections (10). 

Currently, various diagnostic assays for diagnosis of C. *trachomatis* were established that among them the cell culture is suggested as the gold standard method. The cell culture has high specificity but low sensitivity and is available only in some research laboratories. For this reason, other methods for diagnosis of this bacterium were suggested such as enzyme-linked immunosorbent assay (ELISA) and nucleic acid amplification tests such as polymerase chain reaction (PCR). Furthermore, both methods are available in most diagnostic laboratories (11). In detail, ELISA kits mostly use enzyme-labelled antibodies against lipopolysaccharide. Since these antibodies can produce cross- reactions with other chlamydial species, this test may produce false-positive results. Otherwise, ELISA has a lower sensitivity than the cell culture. On the other hand, PCR is a method with high sensitivity and specificity that it's result is not dependent to either viability or an intact state of target organism. Genes targeted for diagnosis of C. trachomatis are MOMP gene, phospholipase gene and 16S and 23S rRNA genes. 

However, PCR has some disadvantages such as the presence of inhibitors with samples and its high costs (12). In Iran, some researchers reported the prevalence of C. trachomatis among women with infertility. Sattari and Badami found that anti- C. trachomatis antibodies in infertile women were significantly more than control group (p<0.05) (13, 14). 

However, to our knowledge, there has not been any adequate research on the detection of C. trachomatis among infertile women in Ahvaz. For this reason, in our study, for the first time, the prevalence of C. trachomatis was compared among infertile and fertile women using PCR and ELISA methods in Ahvaz city, Iran.

## Materials and methods

**Study design**

This case-control study 100 infertile and 125 fertile women age range between 18-49 yr were selected and conducted between August 2016 to January 2017 at the Infertility Clinic of University Jahad, Ahvaz, Iran. According to questionnaire, age, gravidity, previous parities, histories of abortion, post coital bleeding, dyspareunia, abnormal vaginal discharges and ectopic pregnancy were documented for all women.

The inclusion criteria for infertile women were inability in pregnancy despite trying at least one year, a certificate of fertility from men, and lack of antibiotic therapy within 30 days before this assessment. The causes of female infertility were the involvement of ovaries, damages of fallopian tubes or uterus, or abnormality of the cervix. The fertile group was defined as women in third trimester of pregnancy admitted to delivery room. In this group, the exclusion criteria were lack of having a history of infertility and recent antibiotic therapy (15).

**Sample collection and processing**

Vaginal swab were collected by a gynecologist and 2 ml blood were taken and transferred to microbiology laboratory of the medical school. The vaginal swabs were centrifuged at 12000 g for 20 min and their pellets were suspended in 500 ml phosphate-buffered saline. The blood samples were centrifuged at 5000 g for 7 min and then their serums were collected in 1.5 ml microtubes for serology test.

**Serology test for C. trachomatis recognition**

Determination of *C. trachomatis* -specific antibodies (IgG and IgM) in the sera was performed by ELISA assay using commercial kit of Euroimmune (Germany) according to the manufacturer’s recommendations. The cutoff values established by the manufacturer were used for the interpretation of results of the IgM and IgG antibodies. Briefly, an IgM titer with the ratio ≥1.1 was suggested as positive result, 0.8-1.1 as the borderline range result, and under 0.8 as negative result. On the other hand, an IgG titer ≥22 RU/ml was suggested as positive result, between 16-22 RU/ml as the borderline range result, and lower than 16 as negative result.

**Detection of C. trachomatis by PCR**

A volume of 200 µl of the suspended pellet was taken in to a 1.5 ml microtube. DNA was extracted using High Pure PCR Template Preparation Kit (Roche Diagnosis, Mannheim, Germany) according to the manufacturer's procedure. In order to identify C. trachomatis, we performed the amplification of MOMP gene (encoding major outer membrane protein that is conserved in all of C. trachomatis strains). The primers used for PCR were as follows: forward primer: 5’- CCTGTGGGGAATCCTGCTGAA -3’ and reverse primer: 5’- GTCGAAAACAAAGTC ACCATAGTA -3’ which amplified a 144 bp fragment from this gene (16). 

The volume of PCR reaction was 20µL and prepared as follows: 10 μl Master Mix 2x (Ampliqon–Denmark), 0.4pmol/μl of each primer, 5 μl of genomic DNA and distilled water up to 20 μl. The amplification was carried out in a thermal cycler (Eppendrof-Germany). The cycling program was corresponded to 1 cycle at 94^o^C for 5 min, 35 cycles at 94^o^C for 30 sec, 56^o^C for 30 sec and 72^o^C for 30 sec and a final extension cycle at 72^o^C for 7 min. The amplicon of 144 bp was visualized on a 1% Agarose (Sina Clone, Iran) gel stained with safe stain (Sina Clone, Iran). The genomic DNA of C. trachomatis with Accession Number of KX298123.1 was used as positive control.

**Ethical consideration**

The study was approved by the Research Ethics Committee, Ahvaz Jundishapur University of Medical Sciences, Iran (Code no: IR.AJUMS.REC.1395.457). The written informed consent form was completed by each participant. 

**Statistical analysis**

Chi-square and Fishers exact tests were used for comparisons of categorical data using SPSS of software (Statistical Package for the Social Sciences, version 16.0, SPSS Inc, Chicago, Illinois, USA). A p-value <0.05 was considered statistically significant.

## Results

In this study, we collected vaginal swabs from 100 infertile and 125 fertile women. The mean age of infertile and fertile groups were 27.15±4.16 and 28.86±4.32 yr, respectively. Infertility duration among the infertile women was 7.35±5.40 yr and the mean gravidity of fertile women was 1.85± 1.08 yr. 

The data extracted from questionnaires of these two groups is shown in table I. According to this table, 26 (20.8%) fertile women had at least a history of abortion in previous pregnancy and 54 (43.5%) cases had pervious parities. We didn’t see any difference in histories of abortion, post coital bleeding, dyspareunia, abnormal vaginal discharges and ectopic pregnancy between these two groups of fertile and infertile women (p>0.05).

**Figure 1:**
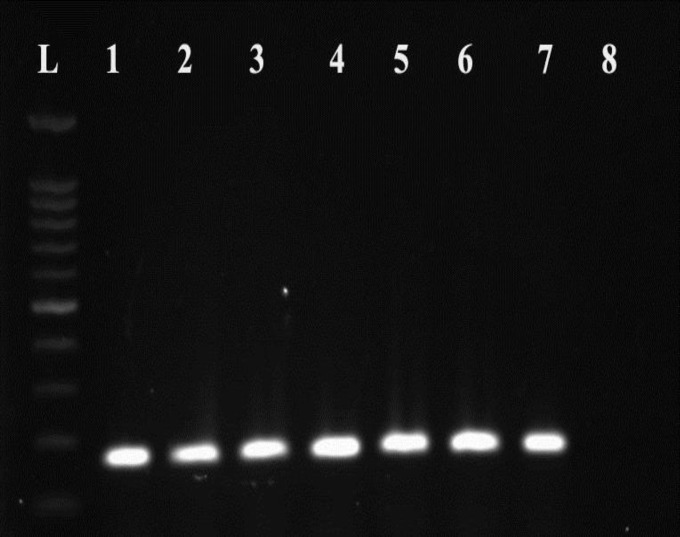
The amplification of *C. trachomatis* by PCR. L: size marker of 100 bp, lanes 1 to 6: Positive samples for C. trachomatis; lane 7: positive control, lane 8: negative control

In our study, the involvement of ovarian (37%) and tubal (32%) factors were the most frequent causes of infertility that followed by uterus (18%), cervix (9%) and others (4%). In our study, 6 (6%) infertile and 2 (1.6%) fertile women were positive for IgM (p=0.21). Also, PCR was positive for C. trachomatis infection in 5 (5%) women with infertility and 2 (1.6%) fertile women (Figure 1). We did not observe any seropositive IgG in both groups and did not find any significant difference between fertile and infertile groups for C. trachomatis infection by PCR or serology (p>0.05).

The positive results of the tests based on causes and types of infertility are shown in table II. We observed among infertile women with ovarian etiology a higher rate of infection cpmpare to the other etiologies. Also, based on PCR and serology assays, C. trachomatis infection was detected only in women with primary infertility.

**Table I:** Data extracted from questionnaires of fertile and infertile women

**Variables**	**Infertility**	**Fertility**	**p-value**
Abortion	29	26	0.45
Post coital bleeding	16	18	0.61
Dyspareunia	14	11	0.48
Abnormal vaginal discharges	52	45	0.36
Ectopic pregnancy	2	1	0.27

**Table II:** Positive results of the tests based on causes and types of infertility

**Variables**		**PCR**	**IgM**
Cause of infertility			
	Ovarian (n= 37)	1	2
	Tubal (n= 32)	3	3
	Uterus (n= 18)	1	1
	Cervix (n= 9)	0	0
	Other (n= 4)	0	0
Types of infertility			
	Primary (n= 82)	5	6
	Secondary (n= 18)	0	0

PCR: Polymerase Chain Reaction

IgM: Immunoglobulin M

## Discussion

Due to the silent nature of *C. trachomatis* infections, most infected women are asymptomatic and therefore remain unrecognized and untreated. There are some evidences that show screening and treating women infected with *C. trachomatis* can reduce PID and its complication (16). 

In our study, the prevalence of *C. trachomatis* among infertile women by PCR and IgM was 5% (5 case) and 6% (6 cases), respectively. Similar to our study, the prevalence of C. trachomatis among infertile women in some regions has been reported such as 52.8% in Brazil by PCR (17), 3.3% in Rwanda by serology (18), and 3.9% in Jordan by PCR (19). This heterogeneity could be due to the diversity in epidemiological condition, the study population, technique used (molecular or serology) or specificity of target primers in molecular methods. 

In our study, the incidence rate of *C. trachomatis* among fertile women using PCR and IgM was 1.6% (2 cases) and 1.6% (2 cases), respectively. Also, we did not find any seropositive IgG in both groups. The low titers or lack of IgG may be due to the absence of previous exposure with *C. trachomatis*. According to results of Malik and colleagues, it seems that IgG detection and past chlamydial infections have a strong role in women with secondary infertility rather than primary infertility (20). 

According to laparoscopy results, the past infections with *C. trachomatis* are associated with a significantly increased risk of tubal infertility in women and these results were confirmed by serology. Furthermore, the severity of tubal damage found in infertile women is directly related to serum antibody titer levels (17). In research of Malik and colleagues IgG antibodies were present in 55% of women with secondary infertility compare to 5.5% in health women (20). In our study since almost infertility cases (82%) was primary, the absence of IgG antibodies can be explained. Also, we showed that the most prevalent etiology of infertility was ovarian defect. 

However, *C. trachomatis* infection was recognized with more frequency in women with tubal defect using PCR and serology. Similar to our study, in study of Rashidi and colleagues, the ovarian defect was reported as the main cause of infertility (15). However, *C. trachomatis* infection was seen significantly in women with ovarian defect (p>0.05). Since in our study, the prevalence rate of *C. trachomatis* infection was lower than study of Rashidi, we can't find an association between *C. trachomatis* infection and infertility causes. So, the selection of a large statistical community for determining this association is essential. 

There are some challenges on the relation of *C. trachomatis* with infertility. Moreover, Malik, Sattari, Badami and Marashi indicated that *C. trachomatis* infection can be as an infertility risk factor. While, Al-Ramahi, Rashidi and Muvunyi didn’t find any significant difference between fertile and infertile women for *C. trachomatis* infection (13-15, 18-21). In our study, using different diagnostic methods, no significant difference was found between these fertile and infertile groups for *C. trachomatis* infection (p>0.05). This difference in the results can be explained due to technique used, the number of study population and type of infertility (primary or secondary). Moreover, similar to our study, in the research of Rashidi the dominant type of infertility was primary and no meaningful relation was found between fertile and infertile groups (15). 

As mentioned above, the past infections with *C. trachomatis* can be a potential factor for infertility especially secondary type that in our work, numbers of the women with this type of infertility was low (18 cases). In the other hand, the techniques used by Malik and colleagues, Sattari *et al* and Badami and salari were based only on serology (ELISA or indirect immunofluorescence) (13, 14, 20). Since serology tests have low sensitivity, for proving and confirming serology results, performing some complementary tests with high sensitivity such as PCR is recommended to these researchers. 

In our study, the frequency rates of abortion, post coital bleeding, dyspareunia, abnormal vaginal discharges and ectopic pregnancy in infertile group were higher than fertile group. However, there wasn’t statistically any significance association between these two groups (p>0.05). Since the prevalence rate of *Chlamydia infections* was low, we can't associated these complications with infertility.

**Limitation**

The main limitations of our study were as followed; 1) the low number of the study population; 2) the low number of women with secondary infertility; 3) short time of study and 4) lacking Real-time PCR.

## Conclusion

In summary, in our study, no significant difference was found between fertile and infertile groups for C. trachomatis infection. Due to the effects of *Chlamydia* infection on fallopian tubes and ovaries, *Chlamydia* screening is highly recommended in infertile women. In our study, ELISA as well as PCR, was recognized as an effective noninvasive test for screening C. trachomatis infections in women suspected of the infection.

## Note

***This article extracted from M.Sc. thesis. (Fatemeh Joolayi)***
